# Relation of Chlamydia trachomatis infections to ectopic pregnancy: A meta-analysis and systematic review

**DOI:** 10.1097/MD.0000000000018489

**Published:** 2020-01-03

**Authors:** Qingchang Xia, Tianqi Wang, Jin Xian, Jingyan Song, Yan Qiao, Zhenni Mu, Honggen Liu, Zhengao Sun

**Affiliations:** a Master of Gynecology in Traditional Chinese Medicine, First College of Clinical Medicine, Shandong University of Traditional Chinese Medicine; b Medical history of Chinese medicine, Institute for Literature and Culture of Chinese Medicine Shandong University of Traditional Chinese Medicine; c Affiliated Hospital of Shandong University of Traditional Chinese Medicine; d Department of Gynecology of Traditional Chinese Medicine, College of Traditional Chinese Medicine; e Master of Gynecology in Traditional Chinese Medicine, First College of Clinical Medicine; f Master of Gynecology in Traditional Chinese Medicine, College of Traditional Chinese Medicine; g Master of Gynecology in Traditional Chinese Medicine, College of Traditional Chinese Medicine, Shandong University of Traditional Chinese Medicine; h Reproductive Medicine Center of Integration of Traditional and Western Medicine, Affiliated Hospital of Shandong University of Traditional Chinese Medicine, Jinan, Shandong, China.

**Keywords:** Chlamydia trachomatis, ectopic pregnancy, meta-analysis, systematic review, the risks of ectopic pregnancy

## Abstract

**Background:**

In a multitude of previous studies, Chlamydia trachomatis (CT) plays an important role in the occurrence of ectopic pregnancy (EP). However, the predictive value of CT infections in the occurrence of EP has not been estimated worldwide. We thus evaluated, by means of a meta-analysis, the current status of the association between CT infections with EP and the potential predictive value of CT infections in EP.

**Methods:**

We evaluated studies performed between the database construction time and August 2018 published in PubMed, the Cochrane Library, EMBASE, and the Web of Science (SCI). The relationship between CT and EP was calculated based upon the predetermined entry criteria for control group selection and the original data. The related articles were analyzed using a random-effects model, and the heterogeneity of the studies was assessed using the I^2^ index. Data were analyzed with the STATA 12.0 software.

**Results:**

Twenty-five studies that recruited 11960 patients were included in the present meta-analysis, and the relation of CT infections with EP were assessed. The association between CT infections and EP risk showed an odds ratio (OR) of 3.03, with a 95% confidence interval (CI) of 2.37 to 3.89. Our results showed that there was a statistically significant difference between the intervention and control groups. The prevalence of CT infections in EP was then calculated by a subgroup analysis: African (OR, 2.22; 95% CI, 1.14–4.31), European (OR, 3.16; 95% CI, 2.10–4.47), North American (OR, 3.07; 95% CI, 1.78–5.31), and Asian (OR, 3.39; 95% CI, 1.95–5.90).

**Conclusions:**

From the results of numerous studies conducted on different continents, this meta-analysis showed a clear association between EP and prior CT infections, that is, CT infections increase the risk of EP occurrence.

## 1 Introduction

An abnormal gestation process by which the embryo implants outside the uterine cavity is defined as ectopic pregnancy (EP). The most common type of EP is tubal pregnancy, which accounts for over 90% of the total cases of EP. The underlying mechanism of EP is exceedingly complex, and the etiology often involves inflammation located at the lumen or the surrounding tissues of the fallopian tubes. After rupture of the infected fallopian tubes, patients often suffer from acute severe abdominal pain, recurrent attacks, vaginal bleeding, or even shock. A recent study showed that there is an increasing trend of the prevalence of EP worldwide.^[1]^ Therefore, EP remains a threat to women's life and health.

Chlamydia trachomatis (CT) is a type of microorganism that is approximately 250 to 450 nm in size.^[2]^ According to the records of the BV System Handbook (1984), CT can be divided into three biologic types: biovar mouse, biovar trachoma, and biovar lymphogranuloma venereum (LGV), with the latter 2 affect humans. At the global level, CT infections are the most common cause of sexually transmitted diseases (STDs).^[3]^ CT primarily infects the epithelial cells of the reproductive tractsand leads to inflammation and tissue damage; and this may be a cause of EP occurrence.^[4]^ CT infections promote inflammatory activities via Chlamydia granules that can cause a medullary immune reaction and inflammation.^[5]^ In women, approximately 40% of the Chlamydia vaginitis affect the endometrium, which cause endometritis and tubal inflammation.^[6]^ Fallopian tube samples obtained from patients with EP are detectable of the deoxyribonucleic acid of c-type trachoma. The frequent association between CT infections and vaginal clue cells or Gram stain abnormalities indicates an overgrowth of anaerobic bacteria, leading to the hypothesis that CT can alter the normal vaginal ecology and cause the complex multi-microorganism infection of the upper genital tracts. Therefore, untreated or poorly treated reproductive tract infections may result in severe long-term reproductive consequences, such as EP.^[7]^ Therefore, CT infections is highly associated with the occurrence of EP to some extent, but the association is still unclear.

During the past decade, several researchers have examined the possibility that CT infections may increase the risk of EP.^[8–10]^ However, other studies have shown that CT infections had a limited correlation with EP.^[11,12]^ Although numerous studies have been performed, there is no meta-analysis with regard for the relationship between CT infections and EP occurrence. In the present paper, we collected the available data and performed a risk assessment using a meta-analysis to investigate the relationship between CT infections and its predictive value for patients with EP.

## 2 Methodology

We used the Preferred Reporting Items for Systematic Reviews and Meta-Analyses statement (PRISMA)^[13]^ guidelines for this study. As this study was a review of published literature, approval of the ethics committee and consent of the patient were not required.

### 2.1 Search strategy

We used 4 search engines, including PubMed, EMBASE, Web of Science, and Cochrane Database of Systematic Reviews, to identify related literature published until August 2018. To thoroughly search the published literature, we used keywords, including ectopic pregnancies, chlamydia trachomatis and pregnancies, ectopic, chlamydia trachomatis and pregnancy, extrauterine, chlamydia trachomatis and extrauterine pregnancies, chlamydia trachomatis and extrauterine pregnancy, chlamydia trachomatis and extrauterine, and chlamydia trachomatis and ectopic pregnancy. We recruited all possible studies, regardless of the main outcome or language. We also used a reference list of key articles published in English and conducted a manual searching.

### 2.2 Inclusion and exclusion criteria

All studies containing keywords in the title or abstract were included in our first lists, and irrelevant articles were eliminated. We deemed the research studies eligible if they were randomized controlled studies entailing CT and EP, with an aim at observing the relationship between CT infections and EP. Exclusion criteria included published reviews, studies without original data, and studies showing associations between CT infections and EP that did not include crucial information, such as the *P value,* OR, and 95% CI.

### 2.3 Quality assessment

All qualified investigation that included analyses of the predictive value of CT infections and EP was performed by Qing-Chang Xia (E-mail: doctoraric@163.com) and Tian-Qi Wang according to the Newcastle-Ottawa Scale (NOS).^[14]^ Studies that contained uncertain data were reassessed by the corresponding author. All data are comprehensively presented in Table 1, including

a) first author, publication year, reference number, study design, case nationality, and number; and
b) the detected samples and main detection methodology.

**Table 1:**
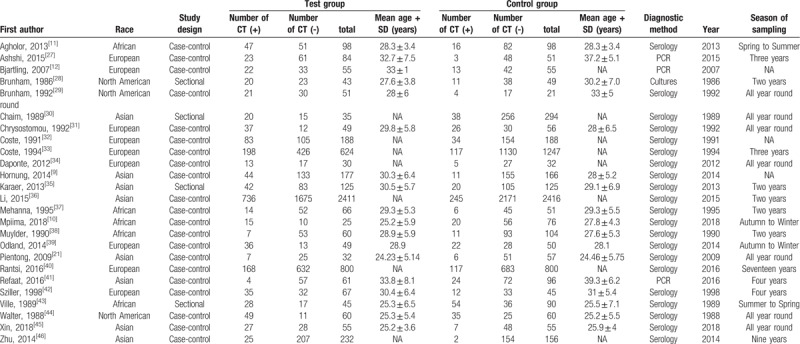
Characteristics of included studies.

### 2.4 Statistical analysis

All analyses were performed using the Stata version 12.0 software. Effect size “OR = 1” indicated that this factor had no effect on the occurrence of disease; OR > 1 indicated that this factor was a risk factor; and OR < 1 indicated that this factor was a protective factor.

### 2.5 Heterogeneity

The random-effects model was applied for the present meta-analysis in terms of the heterogeneity among all studies.^[15]^ To assess heterogeneity, we used the Cochrane Q test and *I*^*2*^ statistics. The heterogeneity of combined OR was tested using the Higgins *I*^*2*^ statistical method (*I*^2^ < 25% indicated no heterogeneity; 25% ≤ *I*^2^ ≤ 50% indicated moderate heterogeneity; and *I*^2^ > 50% indicated strong heterogeneity).^[16]^

### 2.6 Publication bias

To study the publication bias, we used the Begg funnel plot and a 2-tailed *P value* < .05 was considered meaningful.

### 2.7 Sensitivity analysis

In this study, sensitivity analysis was performed to examine the effect of a single study on the combined effect by removing the individual survey. If the estimated value of the point after deleting a study fell beyond the 95% CI of the total effect amount (or was significantly different from the combined effect amount), we considered the study in question to have exerted a great influence on the combined effect amount; and that this study required a further review.

## 3 Results

### 3.1 Eligible studies

**Figure 1:**
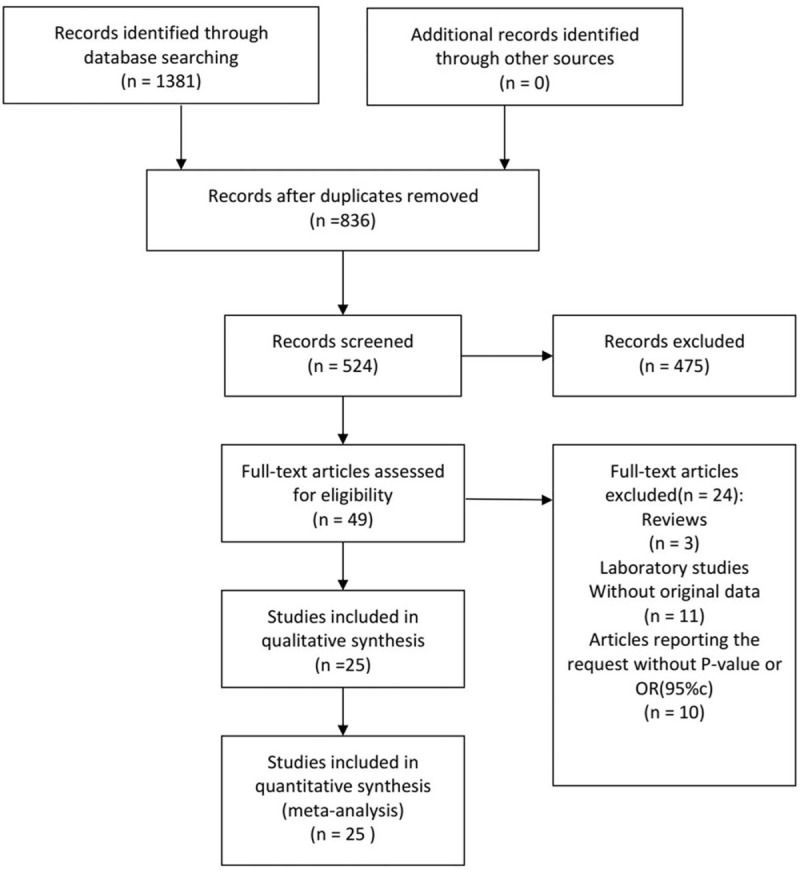
Study flowchart of selected articles for final analysis.

We initially retrieved 1381 studies in our preliminary literature search, and the screening of titles and abstracts resulted in 49 published articles that may have correlation of CT infections with EP. After carefully reading each literature, we only included 25 studies (encompassing 11,960 patients) that met our inclusion criteria. The literature screening process and results are shown in Figure 1, The characteristics of the included studies are shown in Table 1.

### 3.2 Primary outcomes

#### 3.2.1 EP patients with CT infections possess a significant OR

**Figure 2:**
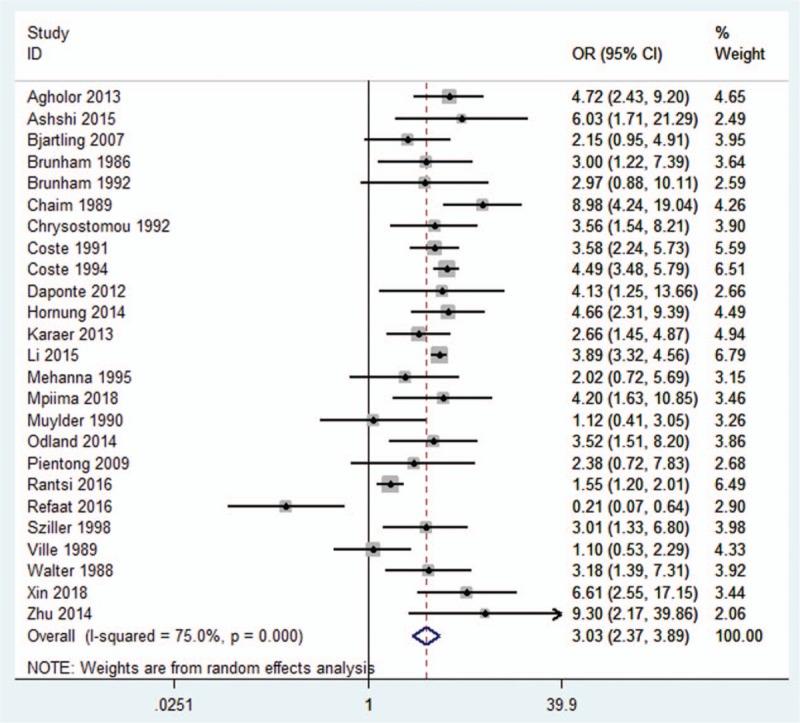
Meta-analysis of the association between CT infections and EP risk.

The results obtained from our studies showed that CT infections increased the risk of EP occurrence (OR, 3.03; CI 95%, 2.37–3.89) with a strong heterogeneity (*I*^*2*^ = 75.0%) (Fig. 2). Additionally, we found that there was a statistically significant relationship between the CT infections and the occurrence of EP. Moreover, our analysis showed that pregnant women with CT infections were more likely to have EP than those who were not infected with CT.

### 3.3 Subgroup analysis

**Figure 3:**
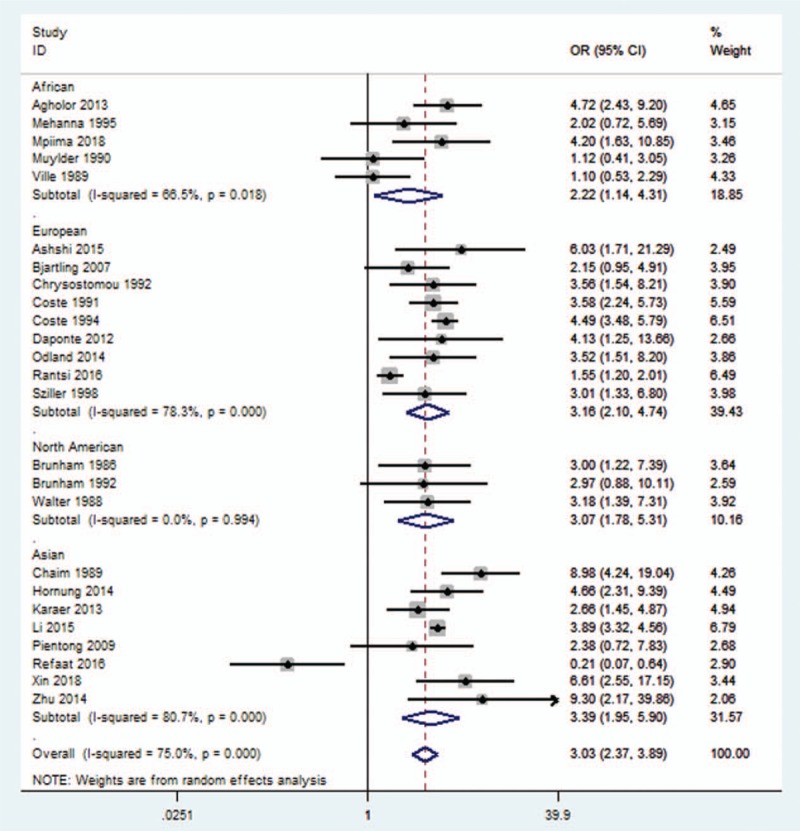
The prevalence of CT infections leading to EP based upon race.

The prevalence of CT infections in EP was calculated by subgroup analysis in different population: African (OR, 2.22; 95% CI, 1.14–4.31), European (OR, 3.16; 95% CI, 2.10–4.47), North American (OR, 3.07; 95% CI, 1.78–5.31), and Asian (OR, 3.39; 95% CI, 1.95–5.90) (Fig. 3).

### 3.4 Publication bias in ORs

**Figure 4:**
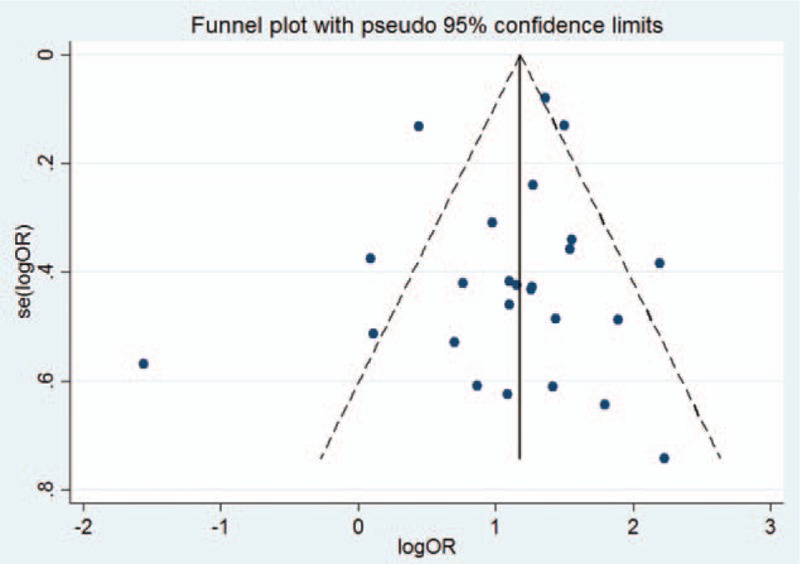
Begg funnel plot for publication bias in the risk-difference analysis.

The Begg funnel plot was used to evaluate the publication bias, and a *P value* of 0.62 indicated that there was no publication bias in the studies that were included in this study (Fig. 4).

### 3.5 Sensitivity analysis in ORs

**Figure 5:**
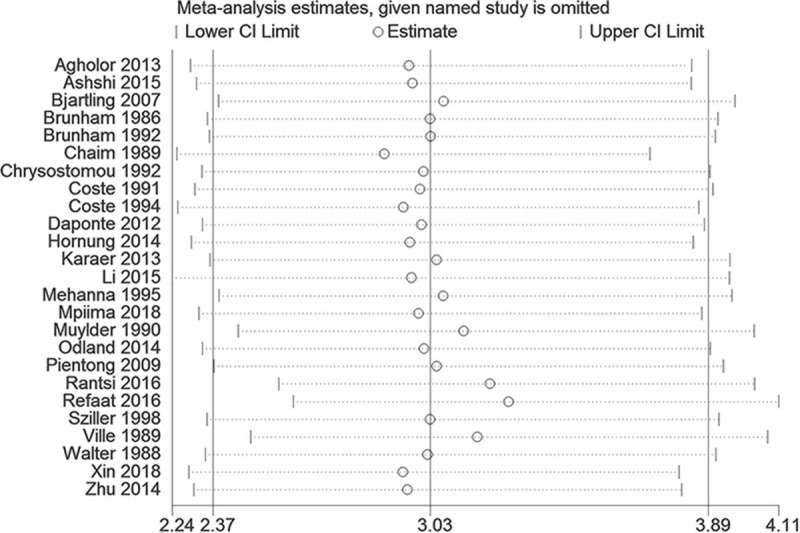
Effects of individual studies on the pooled overall effect.

Our sensitivity analysis did not find any single study that had an impact on the total pooled effect, indicating that no study had a significant impact on OR or 95% CI (Fig. 5).

## 4 Discussion

At present, the exact causes of EP are still unclear; thus, there exists controversy in whether CT infections have a correlation with the occurrence of EP. Additionally, studies regarding the relationship between CT and EP have conflicting results. CT is the most frequently reported pathogen of the bacteria-related sexually transmitted diseases. CT infections can be symptomatic or asymptomatic, and subclinical infections may persist for a longer period of time, which leads to tubal inflammation and symptomatic Chlamydia infection with severe consequences, such as pelvic inflammatory disease and EP.^[17,18]^ The main purpose of this meta-analysis was to elucidate the relationship between CT and EP (OR, 3.03; CI 95%, 2.37–3.89) and determine the predictive value of CT infection for EP. Our results showed that there was a significant relationship between CT infections and EP. Evidence showed that CT infections could be one of the most likely causes of EP. Notably, the infection of CT increases the risk of EP. Additionally, the present subset analysis showed that the prevalence of CT infections and EP was found to be highly correlated in European (OR, 3.16; 95% CI, 2.10–4.47) and Asian (OR, 3.39; 95% CI, 21.95–65.90) women. This prompts us to propose that women with CT infection have a high risk of EP occurrence. Therefore, our study is the first to find a close correlation between CT infections and EP, which may provide insights into the prevention of EP. In this regard, treatment of CT infection could dramatically reduce the risk of EP, and this may have important implications for the safety of many pregnant women.

During the past 20 years, we have seen an expansive analysis of the relationship between CT infections and EP, and several published studies relevant to this research have confirmed that CT infections increased the risk of EP. Shaw et al. proposed that CT can activate tlr-2 in the epithelial cells of the fallopian tubes, giving rise to a plethora of factors associated with embryonic implantation and smooth muscle contraction [e.g., prokineticins (PROK), activin A, and interleukin 1 (IL-1)].^[19]^ Previous studies have shown that a protease of CT (CT441) can interfere with the estrogen-signaling pathway in host cells and induce tubal inflammation,^[20]^ which is a trigger event for EP. Conversely, previous studies performed on EP patients with CT infections had emphasized that CT exerted a limited effect on EP.^[21]^ One study showed that the occurrence of EP was principally related to an abnormal placenta (e.g., “blighted ovum” syndrome and embryonal molar pregnancy) or trophoblastic disease during pregnancy, suggesting that Chlamydia infection played a small role in the pathogenesis of EP.^[21]^ Furthermore, CT infections are not the primary predisposing factor for EP, as 52% of EP patients do not have Chlamydia trachomatis antibodies in their sera.^[22]^

This study analyzed problems in the relationship between CT infections and EP. Our results indicated that CT infections were a significant cause of EP, and infection with CT increased the risk of EP. Therefore, this meta-analysis suggested that the examination of CT should be capture women's attention, especially in those who are pregnant. When women are infected with CT, they should pay attention to the possible occurrence of EP. Therefore, this study has come up with several crucial issues. CT infections may be an important indicator for predicting ectopic pregnancy. In the subgroup analysis stratified by races, we found that the risk of acquiring EP with chlamydia infection was different among women of different races. We thus found that women in Asia and Europe should pay more attention to the infection of CT during their conception.

In this meta-analysis, we included 22 serological studies and 3 PCR studies. However, there is no conclusion on whether the detection method will affect the positive rate of CT, and some researchers believe that serology has a higher detection rate for CT. Therefore, they recommended that serological tests should be performed, while others thought that different detection methods will not affect the results.^[23,24]^ In our study, not all of the three studies we included with the PCR test showing that patients infected with CT had a lower risk of acquiring EP (Bjartling et al, 2007 OR, 2.15;95% CI, 0.95–4.19; Ashshi et al, 2015 OR, 6.03; 95% CI, 1.71–21.29; and Refaat et al, 2016 OR, 0.21; 95% CI, 0.07–0.64). Most likely, these studies indicate that the test method may not be the most critical factor for the results.

The present study did not specifically mention the treatment of CT because our goal was to evaluate the association between CT and EP. It is still unclear whether it can improve the pregnancy outcome after medical treatment of CT. Genc et al, showed that there was no difference in pregnancy outcomes after different treatment of pregnant women infected with CT.^[24]^ While Genc et al agreed that different treatment options had different effects on pregnancy outcomes.^[25]^ To exclude the effect of medical treatment on the results of this study, we did not include RCTs involving medical treatment in the scope of the study. Therefore, no study with medical treatment of CT was included. In addition, since most women infected with CT have no obvious symptoms,^[26]^ many positive cases may be missed, we did not analyze the prevalence of CT.

Several limitations to this meta-analysis should be mentioned, including the specific language used in studies (English) and different types of studies that we included, leading to relatively small sample sizes, different detection methods used. Some of these factors might end up with a large quantity of heterogeneity (*I*^2^ = 75.0%) in this study. Another limitation was the inability to obtained detailed data from studied articles, leading to the exclusion of a large number of articles that could not be processed in meta-analysis though did show significant results on this topic. Therefore, to better understand and utilize the clinical practice of CT in EP, larger-scale prospective cohort studies will be necessary.

In summary, we present the first systematic review with regard for the risk of EP in pregnant women with CT infection in different races. Although large-scale prospective cohort studies are required, our results show a correlation between CT infections and EP and CT infections can increase the risk of EP.

## 5 Conclusion

Although larger-scale prospective cohort studies are required, our study provided an evidence showing that there is a correlation between EP and CT infections. The results obtained from this analysis suggest that pregnant women with CT infections were more likely to have EP than those who were not infected with CT. Therefore, for pregnant women, the healthcare providers should pay more attention to the prevention and treatment of CT infections.

## Author contributions

**Data curation:** Qingchang Xia.

**Formal analysis:** Tianqi Wang.

**Investigation:** Zhenni Mu.

**Methodology:** Tianqi Wang.

**Project administration:** Qingchang Xia.

**Resources:** Jingyan Song.

**Software:** Tianqi Wang, Jingyan Song, Jin Xian.

**Supervision:** Zhengao Sun.

**Validation:** Yan Qiao.

**Visualization:** Honggen Liu, Jin Xian.

**Writing – review & editing:** Qingchang Xia.
